# Rapid Deductive Qualitative Analysis in Implementation Science: A Practical Training Protocol

**DOI:** 10.66815/ajqr/19192

**Published:** 2026

**Authors:** Shelly Palmer, Laura E. Balis

**Affiliations:** Center for Nutrition and Health Impact, Omaha, NE, USA

**Keywords:** Qualitative methods, Rapid analysis, Protocol

## Abstract

Implementation science researchers continue to develop innovative methods to speed the translation of research to practice. One such approach, rapid qualitative analysis, which uses streamlined analytic tools to achieve rigorous results in an abbreviated timeline. While the Planning for and Assessing Rigor in Rapid Qualitative Analysis (PARRQA) framework provides clear guidance for consistently reporting findings, we realized a need for greater training protocols to support team members and answer practical questions. The purpose of this methodological note is to share our teams’ rapid deductive analysis protocol. The includes instructions for 1) developing a coding template that aligns with the interview questions, 2) coding the first interview and refining the template, 3) using a two-coder process to code remaining interviews, 4) using transcripts to select representative quotations, 5) checking quality throughout the process through senior researcher review and meeting debriefs, 6) using a two-coder process to write a summary and select an exemplar quotation for each question/construct, and 7) reporting results. This protocol supported our team in consistently applying rapid deductive approaches to qualitative analysis for deliverable development in a timely manner. Researchers continue to debate whether analysis approaches should be more or less rapid. We recommend using rapid deductive approaches for focused research questions to quickly share findings and inform practice. We encourage other researchers to adopt and adapt our protocol, in conjunction with reporting frameworks such as PARRQA, to continue to advance the field on timely deductive qualitative analysis approaches.

## Background

Implementation science researchers continue to develop innovative methods to speed the translation of research to practice, which traditionally has taken 15 to 17 years (Balas & Boren, 2000; Khan et al., 2021). Approaches that are relevant, rapid, and rigorous, and that do not contribute to translational lag time are emerging (Davis & Beidas, 2021; Peek et al., 2014). One such approach, rapid qualitative analysis, was proposed in in 2013 and has been gaining traction since then (Hamilton, 2013; Hamilton & Finley, 2019; Kowalski et al., 2024). Rapid qualitative analysis is conducted on a shortened timeline and uses streamlined data collection methods that correspond with analysis tools, without compromising rigor (Kowalski et al., 2024). It is recommended for use when 1) the project requires a rapid turnaround of findings to inform the implementation phase, 2) there are multiple data collection time points, such as in a longitudinal study, 3) there is a focused research question, or 4) partners or policy makers have a time sensitive deadline to inform next steps (Kowalski et al., 2024).

As noted by leaders in the movement for rapid deductive approaches, “rapid” does not necessarily mean “easy” (Kowalski et al., 2024). This approach still requires thoughtful design and training team members to ensure consistent data collection, coding, and analysis (Kowalski et al., 2024). The Planning for and Assessing Rigor in Rapid Interview Analysis (PARRQA) framework provides clear guidance for consistently reporting findings from rapid deductive qualitative analysis (Kowalski et al., 2024).

Briefly, the deductive rapid qualitative analysis method detailed in the PARRQA framework begins with developing a data collection instrument following a guiding theory, model, or framework (Kowalski et al., 2024). In implementation science research, this is often a determinant framework, such as Consolidated Framework for Implementation Research (CFIR) (Damschroder et al., 2022), or a planning and evaluation framework, such as Reach, Effectiveness, Adoption, Implementation, Maintenance (RE-AIM) (Glasgow et al., 2019). Additional considerations for selecting a relevant framework and developing data collection tools are outside the scope of this article, but are detailed in other sources (Finley et al., 2022; Gale et al., 2019; Hamilton, 2013; Hamilton & Finley, 2019; Kowalski et al., 2024). Next, researchers develop a coding template in alignment with data collection instrument and selected framework constructs (Kowalski et al., 2024). Flexibility during rapid deductive approaches is maintained throughout data collection as the questions are structured in an open and non-leading way, and during analysis as the coding template is refined to operationalize constructs (Robinson & Bailey-Rodriguez, 2025). Researchers take notes into the template during or directly after each data collection event (Kowalski et al., 2024). After all data is collected, researchers synthesize and summarize data by domains or research questions to compare across cases (Kowalski et al., 2024). Illustrative quotations are added, and results are disseminated through practitioner-friendly methods and/or peer-reviewed manuscripts.

However, after piloting this analysis approach in multiple projects, we realized a need for enhanced training protocols to support team members – both those newer to qualitative analysis and those trained in more traditional approaches such as grounded theory. For example, we found that team members’ data collection notes varied in format (e.g., bullet points vs. complete sentences) and length, which made summarizing difficult. We also realized that we needed a more specific process for identifying representative quotations, as referring back to recordings or transcripts during the summarizing phase was inefficient. We also noticed that we needed to provide more guidance on selecting exemplary quotations, such as how much text to include to illustrate the data.

Previous work has compared rapid deductive methods to more traditional methods and found that the two methods produced consistent results (Brown et al., 2025; Gale et al., 2019; Nevedal et al., 2021). Thus, we did not aim to replicate these studies by comparing the effectiveness of our approach. The protocol has not yet been formally evaluated and its effectiveness should be examined in future research. Instead, the purpose of this methodological note is to expand on the guidelines in PARRQA by describing the rapid deductive analysis protocol we developed to train team members and ensure rigorous methods.

## Rapid Deductive Qualitative Analysis Protocol

This protocol focuses on collecting, analyzing, synthesizing, and reporting interview data through a rapid deductive approach in alignment with the PARRQA (Table 1). The protocol includes instructions for 1) developing a coding template that aligns with the interview questions and constructs, 2) coding the first interview and refining the template, 3) using a two-coder process to code remaining interviews (interviewer codes during/after interview, reviewer listens to recording and edits notes), 4) using transcripts to select representative quotations, 5) checking quality throughout the process through senior researcher review and meeting debriefs (Barnard et al., 2025), 6) using a two-coder process to write a summary and select an exemplar quotation for each question/construct, and 7) reporting results. To train team members, we recommend sharing the written protocol and then conducting a training that includes visual examples, specific team member assignments, and discussion. Of note, our guidance refers to protocols for interviews, as our team primarily conducts interview research; rapid deductive approaches are also appropriate for focus groups and other data collection methods (Kowalski et al., 2024). See table 1 below.

## Data collection and analysis

Prior to beginning data collection, researchers will need to develop a coding template that aligns with the interview questions and constructs of the selected theory, model, or framework. We typically create templates in Excel with a separate tab for each interview participant and a tab for the summaries. Each interview question/construct is entered into a separate row with notes and quotations entered in separate columns.

### Coding the first interview and refining the template

To establish coding consistency, all team members independently code the first interview. Each team member listens to the recording, takes notes, and selects representative quotations, using the process detailed in the following section for coder 1. Next, during a team meeting, coders resolve coding discrepancies and agree on final notes and quotations for each interview question. The team also revises the coding template, if needed, to improve reliability. Once the first interview is coded, all remaining interviews are coded by two team members (herein called coder 1 and coder 2).

### Coding interviews (coder 1)

During the interview, coder 1 takes notes directly in the corresponding participant tab in the coding template. Information that answers the research question should be prioritized and content should be minimally interpreted (i.e., content should be a description of the participant’s responses vs. the researchers’ interpretation) (Kowalski et al., 2024). After the interview, coder 1 revises note as needed to ensures they are concise (e.g., three to five complete sentences) and understandable to someone who was not present during the interview.

### Selecting representative quotations

In the quotation’s column of the coding template, coder 1 uses the transcript to identify and copy one to two verbatim quotations per construct/question. To standardize quotation length, we train coders that each quotation should represent one meaning unit (i.e., words, phrases, or sentences that contain related content that relay one specific thought or idea) (Graneheim & Lundman, 2004). Meaning units are typically no more than two sentences long. We also include the participant identification number at the end of the quotation to show representation from diverse participant voices. Finally, coder 1 ensures the content of the quotation is understandable and can stand alone by adding context in brackets, when needed (e.g., to clarify what “it”, “they”, or “that” refers to).

### Checking coding (coder 2)

We recommend that coder 2 is another team member who was present during data collection or is familiar with the research question. Coder 2 listens to the interview recording and confirms, adds, or removes text to ensure the notes are relevant to the research question, align with the interview question/construct, make sense as stand-alone content, and are concise and written in complete sentences. We recommend making edits in red text and strikethrough to show the reconciliation process and maintain audit trails (Cutcliffe & McKenna, 2004).

### Quality checks and meeting debrief

After each team member with coder 1 responsibilities have coded one interview, the project lead conducts a quality check of each team members’ notes. This entails reviewing notes and quotations, looking for coding inconsistencies, and checking for missed important concepts. The project lead discusses any inconsistencies with the individual coder and provides additional guidance during regular team meetings to address common inconsistencies.

## Data synthesis and reporting

### Developing summaries and selecting exemplary quotations

In the summary tab of the coding template, one of the coders writes a three- to five-sentence summary to combine the findings across all interviewees per construct/question. The summarizer also reviews the selected representative quotations and chooses an exemplary quotation that best illustrates the summarized content. A second team member reviews each summary to ensure it contains content reported across interview notes, is concise, and relates to the interview question/construct and research question. The reviewer also checks the exemplary quotation to ensure it is the best representation of the summary. We also suggest a final check from the project lead to review and finalize all summaries and exemplary quotations. Finally, to ensure quotations are verbatim, we check each exemplary quotation by listening to the interview recording.

### Reporting results

Finally, results are shared through project reports and manuscripts. One approach to sharing results is developing a table with the interview guide constructs, questions, summaries, and key quotations (Table 2). Additionally, some projects require depositing raw data in a repository or submitting with the manuscript as a supplemental file. Prior to this step, we review all interview data to ensure any identifiable information (e.g., names of people or organizations) has been removed and additional context has been added as needed (e.g., to replace “it” or “them”).

When reporting results in peer-reviewed manuscripts, our team has found it useful to include both the PARRQA and the consolidated criteria for reporting qualitative studies (COREQ) checklist (Kowalski et al., 2024; Tong et al., 2007). The COREQ was developed to comprehensively report qualitative studies and includes important considerations that supplement PARRQA. For example, the COREQ includes a “research team and reflexivity domain” that suggests writing a reflexivity statement including researchers’ credentials, occupation, gender, experience and training, prior relationship with participants, and any other relevant characteristics (Tong et al., 2007). See table 2 below.

## Discussion

This training protocol supported our team in consistently applying rapid deductive approaches across nine projects (L. Balis et al., 2024; L. Balis, Palmer, et al., 2025; L. Balis, Stott, et al., 2025; L. E. Balis et al., 2025; Fung Uy et al., 2026; Palmer et al., 2025, 2026). In addition, the protocol was adopted by our colleagues, with five project leads requesting our training protocol and incorporating rapid deductive approaches in their work. This included one study with 11 researchers conducting over 100 interviews (publication in progress), demonstrating the protocol’s utility across large research teams. However, as with other emerging methods, additional questions remain.

One question is related to whether and how saturation (i.e., the point at which new data would provide little to no unique information (Hamilton & Finley, n.d.)) should be assessed. Experts in rapid qualitative research recommend relying on information power instead of saturation, especially while answering complex implementation science question (Hamilton & Finley, n.d.; Malterud et al., 2015). While assessing saturation is not relevant for all qualitative analysis methods (Hamilton & Finley, n.d.; Malterud et al., 2015), we find it useful to calculate for our studies analyzed through rapid deductive analysis, such as for justifying to funders when data collection can cease or explaining to reviewers that we conducted “enough” interviews.

In these cases, assessing saturation has been challenging, as the approach uses a priori theoretical constructs to organize the data instead of approaches such as grounded theory that includes the development of themes. Without themes, typical saturation calculation methods are difficult to use. Our team has used several different methods to overcome this challenge, including a team-based approach to write construct summaries and discuss if saturation had been reached (L. Balis, Stott, et al., 2025), additional thematic analysis to calculate saturation (Fung Uy et al., 2026), and identification of the presence of specific content (e.g., implementation strategies) for saturation analysis (L. Balis et al., 2024). Future research could investigate the best method of assessing saturation, when appropriate, for rapid deductive approaches.

An additional challenge for our team has been determining how to present results from rapid deductive analysis in the publication results section text versus tables. Typically, qualitative studies report the analyzed content in the results paragraphs, although this leaves the publication without a table, and there is value in presenting qualitative data in table format for organization and readability (Cloutier & Ravasi, 2021). For example, when using more traditional approaches (e.g., thematic analysis), we develop a table that reports themes, subthemes, categories, and representative quotations and add detailed descriptions of each in the text (L. Balis et al., 2024). In rapid deductive approaches, the final analyzed summaries and representative quotations are already in table format (e.g., Table 2). This has left us with a challenge of how to represent the results in a table without duplicating the text. We tried several approaches, including abbreviating the content in the table and adding additional distinctions in the table (L. Balis, Palmer, et al., 2025; Fung Uy et al., 2026) (e.g., indicating whether summaries represented barriers, facilitators, or both) (L. Balis, Palmer, et al., 2025). Future work should determine recommendations for content that should be presented in tables and in the text.

A final consideration is whether rapid deductive approaches can be applied in different research settings. For example, an increasing amount of implementation science research is being conducted in low to middle income countries (LMIC) (Le et al., 2022; Ojo et al., 2021; Sivaram et al., 2014), and scholars have investigated the need for methodological adaptation in this context (Means et al., 2020). The balance between adapting existing methods and models and creating new ones is an ongoing challenge in implementation science: using approaches that fit context is crucial, but the desire to develop novel contributions has resulted in a proliferation of models that can prove challenging to navigate (Nilsen et al., 2026; Strifler et al., 2018). In the case of rapid deductive analysis, we recommend starting with a theory, model, or framework that has been used in the setting of interest. For example, CFIR has been adapted for LMICs to include a novel domain and constructs. Other theories, models, and frameworks may not need to be adapted; for example, RE-AIM has been used across multiple countries and settings (Glasgow et al., 2019). Overall, operationalizing the selected theory, model, or framework to the research question is key and rapid deductive approaches allow for this flexibility.

## Conclusions

Interestingly, recent implementation science literature has recently called for both more streamlined rapid methods (e.g., using an abbreviated version of the CFIR with fewer constructs) (Domlyn et al., 2026) and advocated for a return to less rapid, more inductive methods (Nilsen et al., 2026). We recognize that rapid qualitative analysis may not be useful for all studies, and emphasize that selecting the right analysis approach for each study’s goals, resources, and partner needs is key. For example, approaches such as grounded theory or phenomenology are more appropriate when an in-depth investigation of new concepts is needed (Lim, 2025). Rapid qualitative analysis has several strengths, including the timely translation of research into practice, few resources required (i.e., costs of transcription, personnel time, analysis software), and ability to share interim findings with partners while still maintaining rigorous methods (Gale et al., 2019; Nevedal et al., 2021; Taylor et al., 2018). In these situations, we encourage other research teams to adopt, adapt, and improve on our protocol as rapid deductive methods become more widely used.

## Data Availability

Data sharing is not applicable to this article as no datasets were generated or analyzed during the current study.

## Appendix

**Table 1:** Practical Considerations for Rapid Deductive Analysis in Alignment with PARRQA^a^

PARRQA Guidelines	Practical Considerations Included in the Protocol
Summary Template Development Phase	
Develop and pilot test a user-friendly summary template	Train the team members in coding assignments.
Ensure there are cross-references to raw data to support continuous comparison and validation necessary for rapid qualitative analysis	Record main takeaways during data collection. Extract verbatim quotations from transcripts. Confirm verbatim quotations from the recordings and transcripts.
Develop summaries that are accurate and concise, but detailed enough to meet project aims	Write a brief summary (three to five sentences) per construct. Use sentence structure (not bullet points) for the summaries.
Identify training and calibration processes to ensure consistency and accuracy in summaries	Coder 1 records main takeaways during data collection and coder 2 conducts quality check by listening to recordings and reading transcripts. Debrief on data collection during team meetings. Discuss discrepancies between coders during team meetings.
Matrix Analysis Phase	
Plan interview matrix structure to reflect project aims/questions	All team members independently code first interview and refine template, as needed.
Describe use of software for matrix analysis	Construct core notetaking template in Excel with one row per construct/question.
Develop a plan for review throughout matrix analysis	Lead team member conducts a quality check after each team member has been coder 1. Lead team member reviews and finalizes all summaries and representative quotations for the deliverable (final report or manuscript).
Rapid Interview Data Synthesis Phase	
Conduct synthesis that is rigorous and responsive to research priorities	Use a sentence structure for the summaries. One team member writes each summary, a second team member reviews and revises each summary, and the lead team member finalizes each summary. Typical dissemination includes one summary and one representative quotation for each construct/question.

Note.

a The PARRQA checklist contains five phases: rigorous design, semi-structured data collection, summary template development, matrix analysis, and rapid qualitative data synthesis. Rigorous design and semi-structured data collection are outside of the scope of this manuscript.

**Table 2:** Example of a table used to disseminate rapid deductive approach results

CFIR Construct and Related Interview Questions	Summary	Exemplary Quotation
**Complexity** How complicated is the project? How complicated is it to evaluate?	Interventions varied in scope and complexity, from straightforward projects to complex initiatives. Overall, interviewees felt that their grant-funded interventions were not too complex to implement or evaluate.	“These [neighborhood design] projects involve implementation in one way or another. So there is a higher level of complexity with the support we are offering with trying to run the data piece which is straightforward and managing construction documents and supporting and raising additional funding [UHC01].”
Self-efficacy How confident are you that you will be able to successfully evaluate the project?	Most interviewees felt confident in evaluating their interventions; familiar evaluation methods facilitated this confidence. Some grantees noted wanting to learn more about empirical evaluation methods to determine intervention impact.	“Something I’ve thought less about, like how we are measuring the efficacy of our own work. but that I think is really important and I do feel confident in our ability to do that [UHC03].”
Culture How do you think your organization’s culture (general beliefs, values) will affect how you evaluate and share the results of the project?	Grantees described strong organizational cultures facilitated by engaged leadership, reinforcement of organizational values, infusing passion into the work, and pursuing collaborative projects.	“We need to ask our community right?… I think our culture of knowing we don’t know it all… and we have to have humility in that, and we have to truly ask questions. I think that is a part of our culture and I think that that serves as well [UHC06].”
Available resources Do you expect to have the resources you need to evaluate and share the results of your project?	Most grantees described a lack of organizational resources available to support program evaluation and dissemination; challenges included organizational capacity constraints, limited internet access, absence of software, overburdened staff, and lack of funding.	“…as we grew from seven to 60 staff overnight… It was like where the CEO is managing everybody so just trying to identify and have a specific staff who would really lead and guide that team, to make sure that we’re collecting everything that we need to would it been something that would be helpful [UHC08].”
Leadership engagement How involved has leadership at your organization been with the project so far?	The majority of the interviewees shared that leaders in their organizations are engaged and support evaluation and dissemination efforts through engaging with projects, expanding evaluation efforts, and participating in collecting data and disseminating results.	“You can find [our CEO] distributing food, going to talk to neighbors, talking with the staff that manage the program. [UHC03].”
Organizational incentives and rewards How important is program evaluation as part of your job?	Interviewees did not receive special recognition for evaluating projects and disseminating results, and these duties were not included in their performance reviews.	“It’s [program evaluation] not part of anyone’s annual performance review but I think everyone knows we want to grow it as much as possible [UHC05]”
External policies & incentives Are there reporting requirements outside of your organization that influence how you evaluate the project?	Several interviewees mentioned external policies (e.g., government contract and funder reporting requirements) that support evaluation efforts. Some mentioned that these requirements do not specify evaluation measures to be used.	“We always have our government contracts… they will tell us like this is the type of information that we need from you [UHC05].”
Reflecting and evaluating: Evaluation How is success of the project measured?	Multiple evaluation barriers existed, including response rates, participant burden, and coordination with health care partners. Strong partnerships were also described as a facilitator to program evaluation.	“Evaluation is always tough because how do you measure what someone is eating… what does that transfer into numbers? Obviously data is important to get funding [UHC02].”
Reflecting and evaluating: Dissemination How will results of the evaluation be distributed to project partners?	Program results were primarily disseminated through funder reports. Other channels including social media, webpages, newsletters and newspapers, conferences, and in-person visits were used to reach multiple audiences (priority populations and partner organizations). No organization had a dissemination plan.	“That’s [dissemination] a perspective we are really invested in with this thing of sharing the data back with the people we are collecting it from. Is something we care a lot about [UHC01]”

Note.

a This study included n=9 interviews with community-based organizations implementing food access programs (Balis, Palmer, et al., 2025) (reproduced with permission from the authors).
